# Workshop on the Development and Evaluation of Digital Therapeutics for Health Behavior Change: Science, Methods, and Projects

**DOI:** 10.2196/16751

**Published:** 2020-02-26

**Authors:** Alan J Budney, Lisa A Marsch, Will M Aklin, Jacob T Borodovsky, Mary F Brunette, Andrew T Campbell, Jesse Dallery, David Kotz, Ashley A Knapp, Sarah E Lord, Edward V Nunes, Emily A Scherer, Catherine Stanger, William C Torrey

**Affiliations:** 1 Center for Technology and Behavioral Health Geisel School of Medicine Dartmouth College Lebanon, NH United States; 2 Department of Psychiatry Geisel School of Medicine Dartmouth College Lebanon, NH United States; 3 Department of Biomedical Data Science Geisel School of Medicine Dartmouth College Lebanon, NH United States; 4 Clinical Research Grants Branch National Institute on Drug Abuse National Institutes of Health Rockville, MD United States; 5 Department of Psychiatry Washington University School of Medicine St Louis, MO United States; 6 Department of Computer Science Dartmouth College Hanover, NH United States; 7 Department of Psychology University of Florida Gainesville, FL United States; 8 Center for Behavioral Intervention Technologies Feinberg School of Medicine Northwestern University Chicago, IL United States; 9 Department of Psychiatry New York State Psychiatric Institute Columbia University New York, NY United States

**Keywords:** digital health, behavioral health, mobile technology, mHealth, digital interventions, behavior change, behavioral science, addiction, mental health

## Abstract

The health care field has integrated advances into digital technology at an accelerating pace to improve health behavior, health care delivery, and cost-effectiveness of care. The realm of behavioral science has embraced this evolution of digital health, allowing for an exciting roadmap for advancing care by addressing the many challenges to the field via technological innovations. Digital therapeutics offer the potential to extend the reach of effective interventions at reduced cost and patient burden and to increase the potency of existing interventions. Intervention models have included the use of digital tools as supplements to standard care models, as tools that can replace a portion of *treatment as usual*, or as stand-alone tools accessed outside of care settings or direct to the consumer. To advance the potential public health impact of this promising line of research, multiple areas warrant further development and investigation. The Center for Technology and Behavioral Health (CTBH), a P30 *Center of Excellence* supported by the National Institute on Drug Abuse at the National Institutes of Health, is an interdisciplinary research center at Dartmouth College focused on the goal of harnessing existing and emerging technologies to effectively develop and deliver evidence-based interventions for substance use and co-occurring disorders. The CTBH launched a series of workshops to encourage and expand multidisciplinary collaborations among Dartmouth scientists and international CTBH affiliates engaged in research related to digital technology and behavioral health (eg, addiction science, behavioral health intervention, technology development, computer science and engineering, digital security, health economics, and implementation science). This paper summarizes a workshop conducted on the *Development and Evaluation of Digital Therapeutics for Behavior Change*, which addressed (1) principles of behavior change, (2) methods of identifying and testing the underlying mechanisms of behavior change, (3) conceptual frameworks for optimizing applications for mental health and addictive behavior, and (4) the diversity of experimental methods and designs that are essential to the successful development and testing of digital therapeutics. Examples were presented of ongoing CTBH projects focused on identifying and improving the measurement of health behavior change mechanisms and the development and evaluation of digital therapeutics. In summary, the workshop showcased the myriad research targets that will be instrumental in promoting and accelerating progress in the field of digital health and health behavior change and illustrated how the CTBH provides a model of multidisciplinary leadership and collaboration that can facilitate innovative, science-based efforts to address the health behavior challenges afflicting our communities.

## Introduction

Over the last 25 years, scientists in the fields of addiction science and mental health have studied how to enhance treatment outcomes, how to improve the dissemination and implementation of effective treatments, and how to reach a greater proportion of the population experiencing behavioral health challenges. Barriers to success have included substandard screening and identification of cases, limited numbers of trained personnel, high burden associated with training, poor fidelity and integrity of treatment delivery, low pay for providers, high costs for delivering the most effective interventions, stigma and burden associated with seeking treatment, and limited individualization and personalization of treatment.

Broadly speaking, the health care field has integrated numerous advances in digital technology at an accelerating pace to improve health behavior, health care delivery, and cost-effectiveness of care. This area of science, often referred to as *digital health*, has been embraced within the realm of behavioral science, allowing for an exciting roadmap for advancing care by addressing the many challenges to the field via technological innovations [1-4]. Technology-delivered treatments, commonly referred to as *digital therapeutics*, offer the potential to extend the reach of evidence-based interventions at reduced cost and patient burden and to increase the potency of existing interventions. Delivering on these promises has substantial public health implications, given that the majority of people with behavioral health needs are not receiving treatment of any kind, and only the minority of those who do receive treatment are provided with an evidence-based intervention. Multiple studies have demonstrated that treatment models assisted by computer-, Web-, or smartphone-based delivery of evidence-based interventions improve access to care, reduce costs, and improve the efficiency of treatment delivery [1-4]. Intervention models have included the use of digital tools as supplements to standard care models, as tools that can replace a portion of *treatment as usual*, or as stand-alone tools accessed outside of care settings or direct to the consumer. Indeed, digital therapeutics have progressed to such an extent that the US Food and Drug Administration (FDA) recently launched an initiative to incorporate digital health into mainstream medical practice [5]. This significant step acknowledges the potential of and the growing evidence for digital therapeutics. The FDA has further defined a class of digital therapeutics as *mobile medical applications*, software apps that meet the definition of a medical device, that is, performing patient-specific analysis and providing patient-specific diagnosis or treatment recommendations, and at least one mobile app has received FDA permission for marketing [6,7].

However, to further advance the potential public health impact of this promising line of research, several areas warrant development and investigation. Although some digital therapeutics for health behavior problems have been deemed effective, effect sizes to date have been generally small to medium, and treatment mechanisms have been understudied. To address this problem, it is essential to focus more research on identifying and understanding specific mechanisms relevant to health behavior change. Importantly, direct comparisons between digital therapeutics and well-understood traditionally delivered interventions may provide the clues necessary for achieving this goal and developing more potent interventions [8]. Given their *on-demand* availability and opportunities for personalization, digital therapeutics may impact the rate and nature by which health behavior change occurs. An increased understanding of the active ingredients and mediators of outcome from such interventions can greatly enhance future efforts to optimize development efforts. Such progress could move the digital health field beyond direct adaptations of existing face-to-face interventions to better harness the dynamic potential of technology in collecting data about individuals in unprecedented ways (and in real time) and in intervening at the exact moments when individuals may be maximally motivated and receptive [9].

Optimization and acceleration of the development and evaluation of digital therapeutics also require advances in the measures and methods used to study treatment processes, mechanisms, and outcomes. Understanding how an intervention produces changes in cognitive, behavioral, or environmental factors that are then, in turn, causally related to health behavior change (eg, reduction in drug use) allows for the identification of more specific treatment targets, which, in turn, permit refinement and optimization of subsequent iterations of an intervention [8]. Continued development of more granular, objective, and valid measures of important mechanism constructs and processes and treatment outcome variables is also essential for the advancement of health behavior change research and development. Technological and data analytic advances offer exciting new approaches for obtaining and interpreting more meaningful and ecologically valid data that can be readily collected in the natural environment over substantial periods. Finally, the pace of science in the realm of digital health can greatly benefit from explicit methodological frameworks to guide research efforts and the application of innovative experimental designs. Several novel and underused methodological approaches are relevant to developing and evaluating digital therapeutics that can supplement the findings from traditional randomized controlled trials (RCTs) [10-13]. Such approaches include flexible, rigorous, and efficient methods, which can help optimize interventions and bridge the gap between clinical trials and community care.

## Center for Technology and Behavioral Health—Workshop

The Center for Technology and Behavioral Health (CTBH; a P30 *Center of Excellence* supported by the National Institute on Drug Abuse [NIDA] at the National Institutes of Health) is an interdisciplinary research center at Dartmouth College focused on the goal of harnessing existing and emerging technologies to effectively develop and deliver evidence-based interventions for substance use and co-occurring disorders [14]. In total, 3 primary cores, treatment development and evaluation (TDE), emerging technologies and data analytics, and dissemination and implementation, bring together a diverse team with expertise in addiction science, behavioral health intervention, technology development, computer science and engineering, digital security, health economics, and implementation science.

The TDE core of the CTBH organized a workshop, “Development and Evaluation of Digital Therapeutics for Behavior Change: Science, Methods, and CTBH Projects,” which was recently held on the campus of Dartmouth College. This was one of the series of workshops launched by the CTBH to encourage and expand collaboration among Dartmouth scientists and international CTBH affiliates across the diverse disciplines that engage in research related to digital technology and behavioral health. The goals of the workshop were to (1) highlight key conceptual and scientific issues vital to the proliferation of research on digital therapeutics, (2) stimulate new collaborations and scientific developments, and (3) showcase the work of CTBH-TDE core investigators. The workshop theme was the *Science of Behavior Change.* More than 50 faculty, students, trainees, and staff attended this all-day meeting in person, and collaborative partners from a number of US institutions and ETH Zürich’s Center for Digital Health Interventions attended remotely. This paper summarizes the workshop sessions, which addressed (1) principles of behavior change; (2) conceptual frameworks for optimizing applications in mental health, addictive behavior, and health behavior change; and (3) the diversity of experimental methods and designs that are essential to the successful development and testing of digital therapeutics for behavior change. Most sessions also provided examples of ongoing projects focused on identifying and improving the measurement of health behavior change mechanisms and the development and evaluation of digital therapeutics. Our goal here was to use the knowledge gleaned during this workshop to provide a broad overview of the wide-ranging research topics that must be addressed to advance the field of digital health and its impact on health behavior problems and to illustrate how our CTBH model can facilitate progress in meeting the vast health challenges facing behavioral health research and service delivery models.

## Science of Behavior Change

WA, the Director of the NIDA’s Behavioral Therapy Development Program in the Clinical Research Grants Division, provided opening comments and highlighted NIDA’s keen interest in research on behavior change and mechanisms of change in research on addictions. He emphasized how science that targets potential change mechanisms, such as self-regulation, stress, resilience, and intra- and interpersonal processes, can have a ubiquitous impact on our ability to provide more focused and parsimonious treatments, that is, personalized medicine. WA concluded with a discussion of how the integration of technology-based treatments can uniquely target and facilitate change in these mechanisms and thereby advance the development of effective interventions for substance use and associated psychiatric disorders.

As the workshop audience comprised a diverse group of multidisciplinary scientists who affiliate themselves with the CTBH, AB began with an overview of key principles of behavior change, from basic theories of learning (eg, Skinnerian principles of reinforcement) to current behavioral economic concepts that are highly relevant to identifying and targeting effective behavioral health change mechanisms. This presentation illustrated how understanding the basic elements of behavior change principles can be translated into personalized, effective treatment elements. As examples, AB described how novel translations of behavior analytic principles of reinforcement and the construct of temporal discounting have been applied to inform the development of innovative treatment strategies that can enhance treatment outcomes for substance use disorders and other health behavior problems [15-17]. He also shared other heuristic behavioral economic principles that are currently being transformed into tools designed to optimize health behavior change [17] and discussed the many ways that technology can be leveraged to facilitate and accelerate the creation and dissemination of effective behavior change interventions.

JD then initiated a series of presentations that underscored the importance of using diverse and innovative methodologies and experimental designs to advance research on digital technology–based treatments. He introduced and discussed the key role that single-case experimental designs (SCEDs) can play in stage 1 testing of technology-based interventions to promote health behavior [18,19]. He illustrated how SCEDs can rigorously and efficiently answer questions about the preliminary efficacy and acceptability of an intervention. He reviewed the essential methodological elements of SCEDs and used examples from his published research on technology-based methods to promote smoking cessation to illustrate these elements [20]. In addition, he discussed how SCEDs can be employed to evaluate potential mechanisms underlying intervention efficacy. Specifically, mechanisms can be assessed by obtaining a time series of changes in measures of the mechanism construct and the outcome and by experimentally manipulating the mechanism using SCEDs. JD concluded by noting some of the practical and scientific advantages of SCEDs and how their use encourages data intimacy, scientific rigor, and innovation.

CS’s presentation alerted the audience to several emerging research methods and designs that may be particularly useful in evaluating and understanding the impact of technology-based tools on health behavior and that can supplement the findings from RCTs (eg, the Multiphase Optimization Strategy and Sequential Multiple Assignment Randomized Trial [SMART]) [21-23]. CS focused on SMART designs, which involve random assignment of individuals to conditions more than once during a study, based on their response to conditions experienced earlier in the study. As SMART designs involve multiple randomizations over time, they test the impact of applying different intervention approaches at critical decision points based on intervention response. CS presented an example of a recently completed SMART trial for adolescent cannabis use [24]. Using a SMART design, this study tested the impact of an initial intervention (adding working memory training to contingency management [CM] for youth with cannabis use disorder at treatment onset) and an adaptive intervention (switching youth who did not show a positive response after the first month of the intervention to higher magnitude CM incentives) on clinical outcomes. CS summarized how the SMART design fits particularly well with digital interventions because rapid assessment of changing (dynamic) predictors of intervention response and adaptive changes in intermediate outcomes in response to interventions are facilitated by the technology. This effort may lead to technology-based tools that can be readily tailored to optimally meet the needs of an individual [25].

## Mechanism of Action and Measurement

The workshop shifted focus with LM’s presentation on targeting mechanisms of action with digital therapeutics, in this case, self-regulation. She first raised awareness of the ubiquitous contribution and challenges that health risk behavior and poor adherence to medical regimens impart on chronic disease development and its management. Self-regulation, a person’s ability to manage cognitive, motivational, and emotional resources to act in accordance with his or her long-term goals, was introduced as an important causal mechanism of health behavior, and deficient self-regulation was proposed as a key target for interventions addressing health risk behavior in chronic diseases. LM outlined existing but disparate literature that highlights the clear promise of interventions that target self-regulation to improve health and the challenges that must be addressed if we are to most effectively address this transdisease process of self-regulation [12]. Examples were provided of how digital technology and data analytics have created unprecedented opportunities to assess and modify self-regulation and thus accelerate scientific understanding and application. Intensive and continuous individual data collection with mobile devices (or passive sensors and digital footprints of Web-based social media) can provide rich personal and environmental data in real time, which sophisticated data analytics can turn into meaningful insights about individual health and inform person-centered adaptive interventions.

LM discussed an ongoing National Institutes of Health funded *ontology of self-regulation* project that involves the development of optimal measures of self-regulation using the aforementioned digital technology and data analytic methods and evaluation of how the mechanism of self-regulation relates to behavior change across 2 clinical populations (heavy smokers and persons with binge eating disorder) [12]. Participants will use a mobile health self-regulation platform that provides science-based behavior change tools to promote improved self-regulation and health behavior and facilitate real-time data collection.

ES provided more detail on the first stage of this project, that is, the application of data analytics to the development of a momentary measure of self-regulation that can be used to effectively evaluate change constructs in naturalistic settings via mobile devices in trials using digital therapeutics to assess and treat health behavior problems. She described the iterative process and innovative data analytics involved in empirically extracting the most informative items derived from the multitude of items contained within the many existing self-regulation assessments to efficiently capture momentary self-regulation status with a brief measure in longitudinal studies. ES showed data from an initial piloting of this 20-item measure using ecological momentary assessment (3 times per day surveys for 2 weeks). Her team used multilevel factor analysis to select 12 items that represented 4 valid self-regulation subscales (perseverance, self-judgment, sensation seeking, and mindfulness). CTBH affiliates have included this momentary measure to assess changes in self-regulation in 4 clinical studies.

The next 2 presentations illustrated the novel advances in measurement that passive sensing can bring to the study of behavioral health by providing examples of how mobile sensing can be used to identify temporal fluctuations in high-risk emotional states (eg, depression and stress). AC described his ongoing project focused on the high rates of depression and anxiety among college students. His study used passive sensing features of a smartphone and a commodity wrist-worn wearable (Microsoft Band2; Microsoft, WA) to monitor multiple behavioral and physiological correlates of depression over time (eg, activity level, conversation frequency, and sleep duration) [26]. These devices monitored location, phone usage, light and sound detection, activity duration (steps), heart rate, skin temperature, and galvanic skin response over 10 weeks, and text messages prompted collection of weekly patient health questionnaire depression scores and other experience sampling responses. Data analytic models using all these data and pre-post surveys that assessed stress, anxiety, productivity, and other relevant behavior or emotions were combined to determine how the passive sensing measures related to the students’ mental health functioning across the semester.

In an excellent example of cross-discipline collaboration, SL presented results from a proof-of-concept pilot study conducted with members of Dartmouth’s Computer Science and Engineering Sciences Departments to evaluate the reliability and acceptability of a wearable sensor system (called the Amulet Sensor System, a wrist-worn device developed at Dartmouth) to passively identify stress in a college student population. The sensor system included a commodity chest strap heart rate monitor to collect heart rate variability data, an electrodermal activity sensor to assess skin conductance data, and the *Amulet* that acted as a data hub for data from the heart rate monitor. The Amulet included an accelerometer and software to conduct an ecological momentary assessment of stress constructs as well as an *event mark* feature to benchmark high-stress periods. SL discussed results about the feasibility of using commodity products for passive detection of stress and the potential of passive sensing techniques as the first line of assessment to trigger just-in-time interventions [27]. The discussion highlighted the importance and roles of multidisciplinary team of scientists from the fields of behavioral health, computer science, and engineering in the development and evaluation of digital approaches to behavioral health.

## Clinical Application

The workshop then shifted to a more clinical focus as EN discussed the problem of intervention adherence in clinical trials using digital health tools, an issue similar to that which occurs with traditional behavioral and pharmacological interventions. He provided examples about how some technology-based interventions can reduce dropout compared with traditional therapist-delivered interventions for substance use disorders but noted that both approaches still have high rates of attrition. EN highlighted that more attention to increased *production value*, that is, more consumer-friendly user interface and user design, or gamification, could help address this vital adherence issue with behavioral health populations. He provided 2 examples of efforts in this direction, one that included high-quality video demonstrations to enhance development of coping skills in the context of treating substance use disorders [28] and one that is using gamification within a digital therapeutic for treating opioid use disorder [29], but he stressed the need to empirically test the assumption that these innovations positively impact adherence. EN showed data illustrating how a digital therapeutic tool had improved adherence to a buprenorphine medication regimen in the treatment of opioid dependence [30] and concluded with a discussion of other ideas for how technology could enhance treatment adherence, for example, remote camera (smartphone) monitoring and confirmation, facial recognition software combined with pill ingestion, automated messaging, and motivational prompts.

The following series of presentations described ongoing digital health projects of the CTBH faculty and affiliates. Each of them highlighted behavior change methods, experimental design features, and potential next steps in their development efforts. First, MB described the development and testing of a mobile intervention to deliver motivational education for smoking cessation tailored for young adults with serious mental illness. She discussed the importance of reducing and quitting smoking to prevent the disparate chronic diseases in this vulnerable population and how design features of digital therapeutics need to be tailored to the special needs of the clinical population to optimize potential efficacy [31]. Her initial pilot study demonstrated increased quit attempts and increased biologically verified smoking abstinence compared with 2 control groups [32].

Next, WT described an international project called *Detection and Integrated Care for Depression and Alcohol Use in Primary Care*, which is funded by the Research Partnerships for Scaling Up Mental Health Interventions in Low- and Middle-Income Countries (Scale-Up Hubs) program of the National Institute of Mental Health*.* The project seeks to build sustainable research capacity and science-based programs in Latin America while simultaneously creating new knowledge to inform science-based approaches to scaling up mental health implementation research [33,34]. In Latin America, the burden of mental health problems is high, and services for mental health care are low. Expanding access to mental health care in a way that can be quickly scaled and have a substantial impact on the population is a significant global challenge. This project involves training the Latin American primary care workforce to use digital technology to enhance screening and diagnosis and to deliver treatment for depression and substance use via a science-based mobile digital therapeutic tool.

The next set of presentations illustrated how social media can be leveraged for conducting digital epidemiological studies, developing and testing of digital therapeutics, and in-person recruitment for traditional clinical trials. JB reported on a particularly cost-effective means of using social media to recruit adult and adolescent populations with unique characteristics. He highlighted existing literature demonstrating that such methods can be used to recruit clinically relevant populations (eg, persons with depression or HIV, electronic cigarette users, and those with alcohol use problems). He then described a series of studies conducted at CTBH using Facebook advertising mechanisms to recruit large and diverse samples of individuals who use cannabis and discussed the rich clinical epidemiological datasets that were obtained [35,36]. Such studies have provided valuable insights into policy impact, cannabis use phenomenology, and cannabis use benefits and consequences. He further highlighted how social media can also be leveraged to locate individuals interested in receiving treatment and for developing and delivering interventions remotely. AK described her innovative project that used social media, that is, private Facebook groups, to recruit adolescents at risk for anxiety and to remotely elicit their feedback on the design of a digital anxiety intervention [37]. She discussed how the observed active engagement and participation of teens in the Web-based focus group suggest that social media platforms may be an effective tool to engage and elicit feedback from youth in the early stages of the intervention design process.

CS concluded the series of demonstration project presentations by describing her Web-delivered intervention (WebRx) for teens with type 1 diabetes (T1D) and their parents. Across several small iterative pilot studies, she developed a novel multicomponent intervention that targeted adherence to self-management behaviors necessary to manage T1D [38]. Adherence-focused strategies include incentives for youth for objectively defined and tracked adherence behaviors, incentives for parents for daily monitoring of youth adherence, and Web-based health coaching to promote effective use of diabetes device data, and digitally delivered working memory training for the teens. CS described results from an RCT that demonstrated the superiority of WebRx, which engendered higher self-monitoring of blood glucose, better visual-spatial working memory and inhibition, lower hemoglobin A_1c_ levels than those receiving usual care, more frequent parent review of the adolescent’s glucometer, and reduced family conflict [38]. The presentation concluded by focusing on how this highly disseminable intervention focused on promoting the effective use of technology and software to monitor and improve medical outcomes.

## Conclusions and Future Directions

This workshop brought together a collaborative group of junior and senior scientists from a diverse array of disciplines, including clinical and experimental psychology, data science and analytics, psychiatry, computer science, and population health, all interested in the same goal, that is, advancing digital therapeutics to improve health behavior. The presentations and discussions illustrated how scientists across these disciplines can learn from each other and jointly expedite the potential of technological innovation for improving public health and health care and how the science of behavior change is essential for maximizing the impact of this endeavor. Workshop participants endorsed finding additional meaningful ways to bring these seemingly disparate groups of scientists together more frequently. Many acknowledged that this was necessary to accelerate this area of science because of the multitude of phenomena involved in optimizing health and health research, for example, complexity of behavior, behavior change, assessment, measurement, big data analytics, intervention design, experimental design, technology capabilities and limitations, motivation for change, adherence to treatment regimens, and for optimal communication, dissemination, implementation, and sustainability of effective discoveries.

Attendees expressed an eagerness to participate in future workshops or alternative interdisciplinary activities that would better connect them to their colleagues and promote more collaborative project proposals. This workshop, the second of a planned series of 3 CTBH workshops corresponding to the Center’s 3 scientific cores [39], confirmed and clearly illustrated the value of these types of structured activities for fostering greater collaboration among our interdisciplinary faculty and scientists. Complimentary ideas for the future included more in-depth workshops focused on *deep dives* into specific topics, organization of cross-disciplinary student and trainee journal clubs, use of alternative formats for workshops with built in time for small interdisciplinary group discussion, and the continued development of CTBH pilot funding opportunities that require specified cross-discipline collaborations.

In summary, our workshop on the development and evaluation of digital therapeutics for health behavior change showcased the myriad research targets that will be instrumental in promoting and accelerating progress in the field of digital health and health behavior change. The CTBH at the Geisel School of Medicine at Dartmouth College provides a model of multidisciplinary leadership and collaboration that can facilitate innovative, science-based efforts to address the health behavior challenges afflicting our communities by engaging and guiding teams of scientists to conduct research that will provide the knowledge and tools to inform more effective public health programming.

## Abbreviations

CM: contingency management
CTBH: Center for Technology and Behavioral Health
FDA: Food and Drug Administration
NIDA: National Institute on Drug Abuse
RCT: randomized controlled trial
SCEDs: single-case experimental designs
SMART: Sequential Multiple Assignment Randomized Trial
T1D: type 1 diabetes
TDE: treatment development and evaluation
WebRx: Web-delivered intervention
